# Treating myofascial pain with dry needling: a systematic review for the best evidence-based practices in low back pain

**DOI:** 10.1055/s-0043-1777731

**Published:** 2023-12-29

**Authors:** Fabíola Dach, Karen S. Ferreira

**Affiliations:** 1 Universidade de São Paulo, Faculdade de Medicina de Ribeirão Preto, Ribeirão Preto SP, Brazil.; 2 Suroit Hospital, Department of Neurology, Salaberry-de-Valleyfield, Quebec, Canada

**Keywords:** Dry Needling, Myofascial Pain Syndromes, Low Back Pain, Agulhamento a Seco, Síndromes da Dor Miofascial, Dor Lombar

## Abstract

**Background** Myofascial pain syndrome (MPS) is a common source of pain in primary care or pain clinics. There are many different ways to manage and treat MPS, such as physical exercise, trigger points massage, and dry needling.

**Objective** The objective of this overview is to highlight and discuss the evidence-based treatment of myofascial pain by dry needling in patients with low back pain.

**Methods** A systematic review was made based on meta-analysis (MA) and randomized controlled trials (RCTs) related to dry needling treatment for myofascial pain in patients with lumbar pain, published from 2000 to 2023.

**Results** A total of 509 records were identified at first. Seventy were published before 2000, so they were excluded. From the remaining 439 studies, ninety-two were RCTs or MA, of which 86 additional studies were excluded for the following reasons: not related to dry needling treatment (n = 79), not published in English (n = 4), duplicated (n = 1), project protocol (n = 1), and not related to myofascial pain (n = 1). So, this review was based on 4 RCTs and two MA. These studies compared dry needling efficacy to other treatments, such as acupuncture, sham dry needling, laser therapy, physical therapy, local anesthetic injection, ischemic compression, and neuroscience education. Despite outcomes and follow-up period varied between them, they showed that dry needling can decrease post-intervention pain intensity and pain disability.

**Conclusion** Dry needling is an effective procedure for the treatment of myofascial pain in patients with acute and chronic low back pain. Further high-quality studies are needed to clarify the long-term outcomes.

## Resumo

**Antecedentes** A síndrome dolorosa miofascial (SDM) é uma fonte comum de dor em centros primários de atenção à saúde ou nas clínicas de dor. Existem muitas formas diferentes de manejar e tratar a SDM, como o exercício físico, a massagem dos pontos de gatilho e o agulhamento a seco.

**Objetivo** O objetivo desta revisão é destacar e discutir o tratamento baseado em evidências da dor miofascial por agulhamento a seco em doentes com dor lombar.

**Métodos** Foi realizada uma revisão sistemática baseada em metanálises (MA) e ensaios clínicos randomizados (RCTs) relacionados ao tratamento da dor miofascial com agulhamento a seco em pacientes com dor lombar, publicados de 2000 a 2023.

**Resultados** Foram identificados, inicialmente, um total de 509 registos. Setenta deles, publicados antes de 2000, foram excluídos. Dos 439 estudos restantes, 92 eram RCTs ou MA, dos quais 86 foram excluídos pelas seguintes razões: não relacionados a tratamento com agulhamento a seco (n = 79), não publicados em inglês (n = 4), duplicados (n = 1), protocolo de projeto (n = 1) e não relacionados com dor miofascial (n = 1). Assim, esta revisão baseou-se em quatro RCTs e duas MA. Esses estudos compararam a eficácia do agulhamento seco com outros tratamentos, tais como acupuntura, agulhamento a seco “sham”, terapia com laser, fisioterapia, injeção de anestésico local, compressão isquêmica e educação em neurociências. Apesar de os resultados e o período de seguimento variarem entre os estudos, os estudos demonstram que o agulhamento a seco pode diminuir a intensidade da dor pós-intervenção e a incapacidade relacionada à dor.

**Conclusão** O agulhamento a seco é um procedimento eficaz para o tratamento da dor miofascial em pacientes com dor lombar aguda e crônica. Mais estudos são necessários para esclarecer sua eficácia a longo prazo.

## INTRODUCTION

Myofascial pain syndrome (MPS) is a regional pain disorder characterized by the presence of trigger points (TrPs) within muscles or fascia. It is a common source of pain in primary care or pain clinics. Travell & Simons described a trigger point as "A highly irritable localized spot of exquisite tenderness in a nodule in a palpable taut band of (skeletal) muscle." They determined experimentally that trigger point activity could be quantified by measuring these signals with an electromyogram (EMG). 1

The diagnostic criteria are defined by physical examination, including the presence of TrPs, pain upon palpation, a referred pain pattern, and a local twitch response. The prevalence of MPS among patients presenting to medical clinics ranges from 30 to 93%, which may be due to the lack of clear criteria and guidelines for MPS. 2

The IASP (International Association for the Study of Pain) Classification of Pain defines that myofascial pain may better be classified as chronic primary musculoskeletal pain (MG30.02), which can be located in the muscles, bones, joints, or tendons. 3 4 Myofascial TrPs may be implicated in all types of musculoskeletal pain. Chronic primary musculoskeletal pain syndromes are distinguished according to location: upper (chronic primary cervical pain), middle (chronic primary thoracic pain), lower back (chronic primary low back pain), and limbs (chronic primary limb pain). In addition, patients may present with spontaneous or evoked pain in the affected region, accompanied by allodynia. These conditions were formerly named “nonspecific musculoskeletal pain”. 3

A recent discussion concluded that myofascial pain should be considered, primarily, as a nociceptive pain condition. However, it can be present in patients with predominantly neuropathic pain, when associated with radiculopathy, for example, or nociplastic pain, when associated with signs and symptoms of central sensitization, such as allodynia and pain hypersensitivity. Therefore, it was suggested the possibility of subgrouping individuals with myofascial pain into nociceptive, nociplastic, or mixed-type phenotype. 5

Despite the prevalence of MPS, its pathophysiology remains incompletely understood. The internal pathophysiology of TrPs suggests localized tissue hypoxia, increased acute inflammatory cascade (bradykinin, substance P, interleukin-1), and lowered pH (acidosis). 1 The first hypothesis, related to TrPs etiology, describes a mitochondrial dysfunction in the development of TrPs. 3 Recently, Fisher *et al* . analyzed a muscle biopsy in active TrPs to evaluate mitochondrial function. Twenty patients with MPS were included in this study to receive a muscle biopsy (the vastus lateralis was used as the control). The researchers did not observe a difference in mitochondrial function between the affected muscles and control, and they concluded that abnormal mitochondria function does not contribute to TrPs. 6 On the other hand, other studies showed the presence of ragged red fibers near TrPs, 7 and morphological signs of an impaired aerobic metabolism are present in female office workers with trapezius myalgia. 8

Diagnostic criteria have been discussed by a Delphi study, 9 where they proposed the following TrPs diagnostic criteria:

- presence of a hypersensitive spot in the taut band, and
- referred pain sensation elicited by stimulation of the point.

Low back pain is a major health problem and an important cause of medical expenses, absenteeism, and disability. Generally, it can be caused by musculoskeletal or neuropathic (radiculopathy, myelopathy) components. The symptoms of low back pain may be accompanied by the activation of myofascial TrPs in the lumbar and proximal muscles with local or referred pain, even when there is also the presence of neuropathic pain. Thus, in these cases is important to access and treat the myofascial TrPs in addition to the treatment of the neuropathic component. 10

There are many different ways to manage and treat myofascial pain. Some include physical exercise, TrPs massage, hands-on therapy, spray and stretch therapy, dry needling, injections, oral medications, low-frequency laser, botulinum toxin, and other alternative therapies. 11 Treatment of myofascial pain aims to improve the quality of life of patients by reducing its frequency and severity. The pharmacological groups recommended are antidepressants (e.g., amitriptyline, nortriptyline, venlafaxine), anticonvulsants (e.g., pregabalin, gabapentin), muscles relaxants (cyclobenzaprine, tizanidine, baclofen), and botulinum toxin (e.g., onabotulinumtoxin A) ( Table 1 ).

**Table 1:** Myofascial pain treatment modalities

Medication		Physiotherapy	Others
Antidepressants	Amitriptyline	Spray and stretch technique	Dry needling
	Duloxetine		
	Venlafaxine		
Muscle relaxants	Cyclobenzaprine	Hands-on therapy	Infiltration with lidocaine, saline
	Tizanidine		
	Baclofen		
Anticonvulsants	Pregabalin	Massage or ischemic compression	Laser - low frequency
	Gabapentin		Botulinum toxin
			Acupuncture

Dry needling is a technique used to treat myofascial TrPs and consists of the insertion of a fine needle into the muscle, mechanically disrupting muscular tissue, without injection of anesthesia. The physiological mechanism considers that dry needling can restore homeostasis in the myofascial TrPs, increasing local circulation, decreasing local inflammation, and reducing both peripheral and central sensitization to pain. 12 Otherwise, it is a low-cost treatment, with no important side-effects for patients, which is highly indicated for those groups of patients with some restrictions for oral treatment.

Some studies suggest superior outcomes with dry needling when compared to no treatment or sham needling. 12 However, the evidence is low-quality and the long-term benefit of dry needling is currently lacking. Considering this, the objective of this overview is to highlight and discuss the evidence-based treatment of myofascial pain in patients with low back pain using dry needling.

## METHODS

### Eligibility criteria

Meta-analysis (MA) and Randomized Controlled Trials (RCTs) related to myofascial pain treatment in patients with lumbar pain, published between 2000 and 2023, were included in this review.

Articles published before 2000 were excluded because we aimed to focus on the most recent techniques, treatments, and clinical outcomes, and the literature has changed significantly over the last 23 years. However, MAs included here are also considered the most important papers before 2000.

Reference lists of articles eligible for full-text review were additionally searched for potentially relevant studies.

### Search strategy and data collection

An electronic search was performed for studies published between 2000 and July of 2023 in PubMed/Medline and PEDro (Physiotherapy Evidence Database), related to the treatment of myofascial pain in patients with lumbar pain. Medical Subject Heading terms and free text terms were collated using the following search terms: (“myofascial”[All Fields] AND (“pain”[MeSH Terms] OR “pain”[All Fields]) AND (“low back pain”[MeSH Terms] OR (“low”[All Fields] AND “back”[All Fields] AND “pain”[All Fields]) OR “low back pain”[All Fields] OR (“lumbar”[All Fields] AND “pain”[All Fields]) OR “lumbar pain“[All Fields])).

The investigators assessed research titles and abstracts to determine their potential eligibility. The same investigators examined the full-text articles of potentially relevant studies to verify if eligibility criteria were met, and to evaluate whether the results were adequately reported. Only MA and RCTs, published in English, and related to the treatment of myofascial pain with dry needling in patients with lumbar pain were considered.

## RESULTS

**Figure 1:**
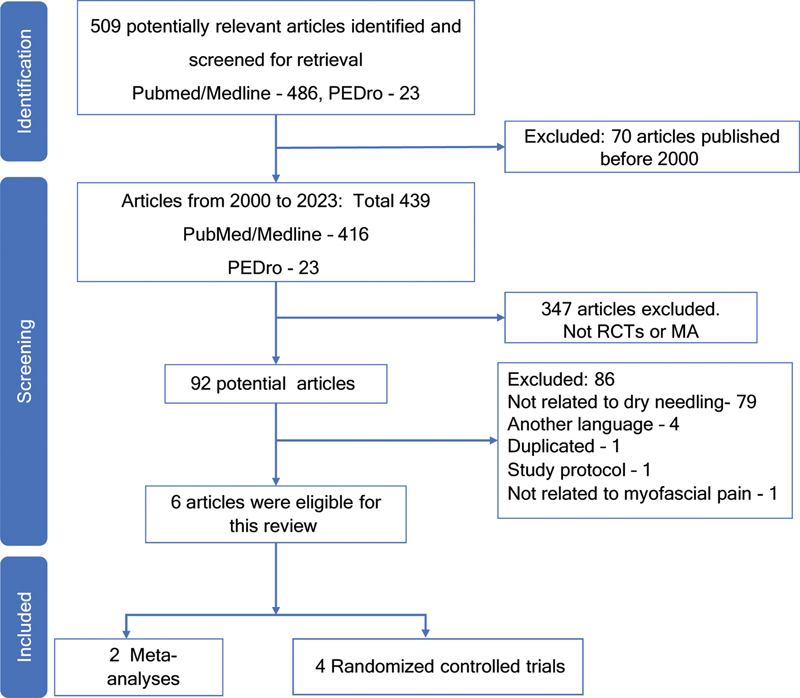
PRISMA 2020 Flow diagram of the study selection process.

The selection of studies is described in Figure 1 . Initially, a total of 509 records were identified, of which 70 studies were excluded for being published before 2000. From the remaining 439 studies, ninety-two were RCTs or MA, of which 86 additional studies were excluded for the following reasons: not related to dry needling treatment (n = 79), not published in English (n = 4), duplicated (n = 1), project protocol (n = 1), and not related to myofascial pain (n = 1). This review evaluated a total of 6 studies, two MA and four RCTs.

### Meta-analysis

Table 2 shows the number of studies, the sample size, and the results of the MAs considered in this review.

**Table 2:** Meta-analysis studies on dry needling in myofascial lumbar pain

Authors (year)	Number of studies	Sample size	Results (Std. Mean difference, 95% CI)
Liu, L; et al. (2018) 13	11	802	**Dry needling versus other treatments** Post-intervention pain intensity: -2.74 [-3.77,-1.71]* Post-intervention pain disability: -0.76 [-1.46,-0.06]* Follow-up pain intensity: -0.43 [-1.17,0.30] Follow-up pain disability: -0.20 [-0.80,0.40] **Dry needling versus Dry needling plus other treatments** Post-intervention pain intensity: 0.83[0.55,1.11]* Post-intervention pain disability: 0.13[-0.14,0.40]
Hu, HT; et al. (2018) 14	16	1233	**Dry needling versus sham needling** Post-intervention pain intensity: -1.06 [-1.77,-0.36]* Post-intervention functional disability: -1.70 [-2.59,-0.81];* Follow-up pain intensity: -1.05 [-1.70,-0.40]* Follow-up pain disability: -0.58 [-1.19,0.04] **Dry needling versus acupuncture** Post-intervention pain intensity: -0.96 [-1.80,-0.12]* Post-intervention functional disability: -0.63 [-0.09, -0.26];* Post-intervention response rate: 1.07[1.01,1.14]* Follow-up pain intensity: -0.47 [-1.04, 0.09] Follow-up functional disability: -0.10 [-0.65,0.45]

Note: *p < 0.05.

The study published by Liu et al. 13 analyzed data on the post-intervention and follow-up effectiveness of dry needling alone versus other treatments for TrPs associated with lumbar pain. A total of 11 studies, published between 2004 and 2016, were considered in quantitative analyses. The sample was composed of 802 patients (56.3% men) with acute (2 RCTs) or chronic pain (9 RCTs). Dry needling was compared to the following treatments: superficial dry needling, acupoint acupuncture, TrPs sham dry needling, super laser therapy, tender points needling, standard physical therapy, local anesthetic injection, and dry needling plus neuroscience education. During post-intervention period, the pain intensity was analyzed through ten studies (673 patients), which pooled results in the random effect models showed that patients who received dry needling had lower pain intensity (-1.06[-1.77,0.36] p = 0.003). The pain disability was analyzed throughout 8 studies (353 patients), and also showed that dry needling patients had lower pain disability compared to other treatments (-0.76[-1.46,-0.06] p = 0.03). The efficacy of dry needling compared to dry needling plus other treatments was analyzed based on 2 studies. The pain intensity analysis favored the association of treatments (0.83[0.55,1.11] p < 0.00001), however, there were no differences between these interventions in relation to pain disability (0.13[-0.14,0.40] p = 0.36). During the follow-up period, the analyses of six studies (213 patients) were considered in relation to pain intensity (-0.43[-1.17,0.30] p = 0.25) and pain disability (-0.20[-0.80,-0.40] p = 0.51), and for both variables, there were no differences between the treatments. 13

Hu et al *.* 14 published an MA to evaluate the efficacy of dry needling in the treatment of lumbar pain through the analysis of 16 studies (1256 patients), published from 1989 to 2015. Dry needling was compared to the following treatments: acupuncture, wrist-ankle acupuncture, sham needling, trigger point injection therapy, laser irradiation, dry needling plus neuroscience education, dry needling in combination with acupuncture, and standard physical therapy. The intervention duration varied from 1 day to 4 months, the number of sessions from 1 to 20, and the follow-up period from 7 to 60 days. However, the MA calculations were conducted only between dry needling versus sham needling or acupuncture. The comparison of dry needling with sham needling was based on two RCTs (43 patients) and showed that post-intervention pain intensity (-1.06[-1.77,-0.36] p = 0.003), post-intervention functional disability (-1.70[-2.59,-0.81] p = 0.0002) and follow-up pain intensity (-1.05[-1.70,-0.40] p = 0.02) favored dry needling. Furthermore, there were no differences between treatments in the follow-up pain disability (-0.58[-1.19,0.40] p = 0.06). When dry needling was compared to acupuncture, at post-intervention period, the effect of dry needling was significantly superior in the reduction of pain intensity (-0.96 [-1.80,-0.12];p = 0.03) and functional disability (-0.63 [-0.09, -0.26];p = 0.0007), as well as in the response rate (1.07[1.01,1.14];p = 0,03). However, there were no differences between groups regarding follow-up pain intensity (-0.47 [-1.04, 0.09];p = 0.10) and follow-up functional disability (-0.10 [-0.65,0.45];p = 0.72). 14

### Randomized controlled trials

Table 3 shows the study population, site of intervention, end-points, and results of the RCTs considered in this review.

**Table 3:** Randomized controlled trials on dry needling in myofascial lumbar pain

Authors (year)	Study sample (n)	Site of intervention	End-points	Results (Std. Mean difference, 95% CI)
Télles-García, M et al. (2015) 15	Mechanical low back pain CG – Dry needling (6) SG – Dry needling plus neuroscience education (6)	Active TrPs in gluteus medius and quadratus lumborum muscles	Baseline versus 3 week scores: ● Pain intensity (NPRS) ● Disability (ODI, RMQ) ● Kinesiophobia (TSK) ● PPT (Algometry on C5-C6, transverse process of L3, second metacarpal, tibialis anterior)	**Within-group change scores** Pain intensity= CG: -3.6 (-6.0, -1.1); SG: -4.2 (-6.6,-1.7) ODI= CG:-24.0 (-39.1, -8.9); SG: -19.5 (-29.9, -9.0) RMQ= CG: -6.1 (-8.0, 4.3); SG: -9.7 (-13.6, -5.1) Kinesiophobia= CG: -5.0 (-11.6, 1.6); SG: -17.7 (-25.1, -10.2) **Between-group difference in change scores** Pain intensity= 0.6 (-3.8, 4.8); ODI= 4.5 (-11.3, 20.4) RMQ= 3.6 (-1.0, 7.2) Kinesiophobia= -12.7 (-21.3, -4.0);* PPT C5-C6 dominant side= 18.0 (-30.0, 65.7) PPT C5-C6 non dominant side= 6.6 (-12.0, 15.6) PPT L3 dominant side= 61.8 (4.5, 128.1)* PPT L3 non dominant side= 58.7 (50.9, 66.5)* PPT 2 ^nd^ metacarpal dominant side= 41.9 (-16.7, 75.3) PPT 2 ^nd^ metacarpal non dominant side= 5.4 (-14.6, 25.8) PPT tibialis anterior dominant side= 8.1 (-13.8, 30.1) PPT tibialis anterior non dominant side= 37.1 (-26.3, 100.5)
Wang, G et al. ( 2016) 16	Chronic lumbar myofascial pain Needle diameters: A – 0.25mm (16) B – 0.5mm (15) C – 0.9mm (15)	Lumbar TrPs	Baseline versus 3-month scores: Pain intensity (VAS) Health survey (SF-36)	**Within-group change in mean:** Pain intensity: A: -2.48*; B: -2.87*; C: -4,43* SF-36: A: 13.31**; B: 14.20*; C:20.14* **Between-group difference mean:** Pain intensity: A: 4.15 ± 2.37; B: 4.05 ± 2.51; C: 2.16 ± 1.64; A versus B, p = 0.858; A versus C, p = 0.064; B versus C, p = 0.047. SF-36: A: 66.00 ± 9.20; B: 67.33 ± 11.36; C: 71.27 ± 7.81. A versus B, p = 0.739; A versus C, p = 0.487; B versus C, p = 0.721.
Castro-Sanchez, AM et al. (2017) 17	Fibromialgia CG – Cross Tape Therapy (32) SG – Dry Needling + Compression for 15 sec. (32)	Active or latente TrPs in latissimus dorsi, iliocostalis, multifidus, and quadratus lumborum muscles	Baseline versus 1 month scores: Pain intensity (VAS) PPT (Algometry) Spinal Mobility (SMS)	**Within-group change in mean:** Pain intensity: CG: 0.56 (1.11, 1.01)* SG: 3.31 (2.50, 4.13)* **Between-group change in mean:** Pain intensity: SG > CG (F = 36.285)* PPT = latissimus dorsi muscle (right axillary portion: F = 9.80)*; multifidus muscle (right L2 level: F = 11.80)*; quadratus lumborum (right lateral superficial upper: F = 6.67)*; quadratus lumborum (right lateral superficial lower: F = 5.38)*. SMS= thoracic spine segmental amplitude in the erect standing position (F = 7.33)*, lumbar spine segmental amplitude in the erect sitting position (F = 11.60)*, sacral slope in the flexed sitting position (F = 5.54)*, the range of flexion for thoracic-lumbar spine segmental amplitude in the sitting position (F = 9.11)*, the range of erect to flexion for the sacral slope (F = 4.48)*, thoracic spine segmental amplitude (F = 6.45)*, and thoracic-lumbar spine segmental amplitude in the sitting position (F = 13.11)*
Álvarez, SD et al. (2022) ^18^	Non-specific low back pain CG – Ischemic Compression (40) SG – Dry needling (40)	Latent trigger point in the gluteus medius muscles	Baseline versus post-intervention, 48 h, and 1 week scores: Pain Intensity (VAS) Quality of life (OQL) Range of motion (SRM) PPT (Algometry on gluteus medius muscle)	**Within-group change in mean (Post-intervention)** Pain intensity= CG: -4.2*; SG: -0.63 PPT= CG:0.66 *; SG: -0.46* **Within-group change in mean (at 48 h)** Pain intensity= CG: -3.27*; SG: -3.0* PPT= CG: 0.37; SG: 0.32* **Within-group change in mean (at 1 week)** Pain Intensity= CG: -3.07*; SG:-3.40* SRM= CG:-0.20; SG:-0.13 OQL= CG:-2.1*; SG:-5.4* PPT= CG: 0.2; SG: 0.32* **Between group differences (at 1 week/mean ± standard deviation)** Pain Intensity= CG: 5 ± 1.81; SG: 4.20 ± 1.26* PPT= CG:4.81 ± 1.18; SG:4.787 ± 0.92 SRM = CG:0.07 ± 0.25; SG:0.07 ± 0.025 OQL= CG:20.73 ± 4.78; SG:15 ± 4.17 *

Abbreviations: CG, control group; NPSR, Numerical Pain Rate Scale; ODI, Oswestry low back pain disability index; OQL, Oswestry quality of life questionnaire; PPT, Pressure Pain thresholds; RMQ, Rolande-Morris disability questionnaire; SG, study group; SMS, Spinal Mouse System; SRM, Schober range-of-motion test; TrPs, trigger points; TSK, Tampa scale of kinesiophobia; VAS, visual analog scale. Note: *p < 0.05.

A small sample RCT investigated the effects of the combination of dry needling and neuroscience education in patients with chronic low back pain. The targets of dry needling were active TrPs at gluteus medius and quadratus lumborum muscles, bilaterally, one session per week for three weeks. The neuroscience education consisted of individual face-to-face sessions focused on education of pain neurophysiology, the role of beliefs and attitudes on patients' pain, as well as patients were encouraged to ask potential questions and to do homework between sessions. The pain intensity was calculated by numbered pain rating scale (NPRS), the disability by Oswestry low back pain disability index (ODI) and Rolande-Morris disability questionnaire (RMQ), kinesiophobia by Tampa scale of kinesiophobia (TSK) and pressure pain threshold by algometry at the transverse process of L3, second metacarpal and tibialis anterior. In both groups, there were improvements in pain intensity and disability by ODI scale. The disability by RMQ and the kinesiophobia (pre- versus post-intervention) were reduced only in the group of neuroscience education. The comparison between groups showed that kinesiophobia and pressure pain thresholds at L3 (dominant and non-dominant sides) were superior in the neuroscience education group, with no differences in other variables. 15

A second RCT focused on the influence of three-needle diameter in the treatment of myofascial pain in patients with chronic lumbar pain. The target of dry needling was lumbar TrPs, the acupuncture needle diameters were 0.25 mm (group A), 0.5 mm (group B), and 0.9 mm (group C), and the analysis was based on pain intensity and health status, which was measured with SF-36. The procedure was made once, with an identical technique, using 20 needles per patient, and the main evaluation was made 3 months after the procedures. Within-groups analyses showed that there was a significant reduction in the intensity of pain in all groups, however, a greater improvement was observed in group C (A: 4.15 ± 2.37; B: 4.05 ± 2.51; C: 2.16 ± 1.64). The comparison of the differences between groups at the 3-month analysis showed that there was a difference between groups A and C, favoring group C. Regarding overall health status, all groups showed significant improvement in longitudinal analyses, and no differences between groups were observed (A: 66.00 ± 9.20; B: 67.33 ± 11.36; C: 71.27 ± 7.81), however, better results also occurred in group C. A complementary analysis showed that the pain intensity of needling, as expected, was significantly greater with the 0.9 mm needles, despite that, patients of all groups were willing to accept the treatment again. 16

The effect of dry needling plus TrP compression versus cross-tape therapy in patients with fibromyalgia was also investigated. The targets of dry needling were active or latent TrPs in latissimus dorsi, iliocostalis, multifidus, and quadratus lumborum muscles. All interventions were made once a week for a month, and the considered variables were pain intensity, pressure pain threshold (PPT), and spinal mobility. After one month of treatment, pain intensity and pressure pain threshold for most TrPs reduced significantly in both groups, being greater in the dry-needling group [dry needling: 3.31 (2.50,4.13 p < 0.01); cross tape: 0.56 (1.11, 1.01) p < 0.016]. The comparison between groups showed a significant reduction in PPT in latissimus dorsi (axillary portion), multifidus, and quadratus lumborum muscles also favoring the dry-needling group. In relation to spinal mobility, the between-groups analyses showed significant greater values in needling group for the following segments: thoracic spine segmental amplitude in the erect standing position (F = 7.33, p= 0.009), lumbar spine segmental amplitude in the erect sitting position (F = 11.60, p = 0.001), sacral slope in the flexed sitting position (F = 5.54, p = 0.022), the range of flexion for thoracic-lumbar spine segmental amplitude in the sitting position (F = 9.11, p = 0.004), the range of erect to flexion for the sacral slope (F = 4.48, p = 0.039), thoracic spine segmental amplitude (F = 6.45, p = 0.014), and thoracic-lumbar spine segmental amplitude in the sitting position (F = 13.11, p =0.001). 17

The study of Alvarez et al. 18 aimed to assess the effectiveness of dry needling versus ischemic compression for the treatment of patients with non-specific low back pain. The targets of the interventions were latent TrPs in the gluteus medius muscle, and the post-intervention analyses were made immediately after the procedure, 48 h and 1 week later. The variables were based on the evaluation of quality of life, range of motion, pain intensity, and PPT. Longitudinal analysis showed that both groups had a significant improvement in pain intensity in all periods, except for the needling at immediate period evaluation, in which differences were not observed. Moreover, there were significant differences between groups in pain intensity, favoring dry needling. However, as the ischemic group had significantly greater VAS (visual analog scale) values pre-intervention, this result should be taken with caution. The between-group analysis also showed no differences in PPT and range of motion (Shober test). In relation to quality of life, both groups showed improvement in scores on the Oswestry questionnaire, however, one week after the procedure, quality of life improved significantly in the needling group in relation to the ischemic group. 18

## DISCUSSION

### Main findings

Based on the studies of this review, dry needling is an effective procedure for the treatment of myofascial pain in patients with acute and chronic low back pain. In general, its effect on the improvement of pain intensity is superior to other treatments.

The main findings of this review were:

- Dry needling can decrease post-intervention pain intensity and pain disability when compared to other treatments 13 14 16 17 18 ;
- The results vary at follow-up 13 14 ;
- Combination of dry needling plus other treatments can be better than dry needling monotherapy 13 ;
- Larger needles are more effective when compared to fine needles. 16
- Dry needling can increase spinal mobility measures 17 and quality of life after treatment. 18

The results of the current systematic review suggest that dry needling is effective in treating myofascial low back pain when compared to other treatments (superficial dry needling, acupuncture, sham dry needling, laser therapy, tender points needling, physical therapy, local anesthetic injection, ischemic compression, and dry needling plus neuroscience education). 13 14 16 17 18 These findings are in agreement with those of previous reviews, 12 19 20 21 which showed that dry needling, for myofascial pain, can be superior to no treatment or sham needling in reducing pain. Other studies showed a lack of evidence related to dry needling directly into myofascial TrPs to recommend as an effective treatment. Anyway, it is difficult to compare these results because they were not related to low back pain specifically. 22 23 24 25

Regarding follow-up and long-term outcomes, it is not clear if the dry needling effect can be long-lasting. 13 14 The outcome varies at follow-up. Liu et al. 13 described no differences between treatments at follow-up, and Hu et al. 14 described a reduction in follow-up pain intensity favoring dry needling (compared to sham needling), but no differences when compared to acupuncture.

Furthermore, results showed new findings from those previous reviews. First, dry needling can be more effective for post-intervention pain intensity when used with other treatments (such as physical therapy and neuroscience education). 13 However, the combination of dry needling plus other treatments did not differ regarding pain disability. 13

Second, for quality of life and functional outcomes, there are studies describing better scores for patients treated with dry needling. 13 18 Also for spinal mobility measures, one study showed greater values in the dry-needling group (compared to cross-tape therapy) for some segments as thoracic and lumbar spines. 17 On the other hand, previous meta-analysis described that there was no effect of dry needling compared to other treatments for functional outcomes. 12

Third, larger needles can be more effective for decreasing pain intensity after treatment. 16 Larger needles are more effective for pain intensity after 3 months of treatment when compared to thin needles. In terms of quality of life (SF-36), both larger and thiner needles are effective. 16

Although low back pain tends to improve spontaneously over time, dry needling may be a useful adjunct to other therapies to reduce pain intensity, pain disability, medical expenses, and absenteeism. Studies with higher methodological quality are needed to clarify some points, especially about long-term outcomes.

### Strengths and limitations

This study reviewed the most important papers about dry needling for myofascial TrPs in patients with low back pain. However, it did not execute a systematic approach before 2000, a meta-analysis was not performed, and it focused on treatment outcomes. On the other hand, meta-analyses described here also reviewed papers before 2000. In addition, there was also a considerable amount of clinical and methodological heterogeneity across studies, which can influence the results.

### Clinical implications

It is hypothesized here that dry needling can be used as a potentially effective and minimally invasive treatment for myofascial pain. It is also a low-cost treatment with no important side effects, which is a special advantage for those patients with some restrictions for oral treatment, such as polypharmacy, association of comorbidities, painkillers overuse, and opioid dependence.

### New directions and future studies

Finally, new foundations guide us towards the development of individualized treatments, including pharmacological and non-pharmacological approaches, such as dry needling. Further high-quality studies with long-term outcomes are needed to clarify the long-term effectiveness of dry needling.

In conclusion, dry needling can decrease post-intervention pain intensity and pain disability for myofascial pain in patients with low back pain when compared to other treatments, such as acupuncture, sham dry needling, laser therapy, physical therapy, local anesthetic injection, ischemic compression, and neuroscience education. The outcomes varied at follow-ups, and new future studies are needed to clarify the long-term effectiveness of dry needling.
